# Pioglitazone Attenuates Sepsis-Associated Acute Kidney Injury by Modulating TLR-4/NF-κB Signaling and Improving Survival and Renal Function

**DOI:** 10.3390/jcm15062270

**Published:** 2026-03-17

**Authors:** Nadir Adnan Hacım, Ahmet Akbaş, Bakiye Akbaş, Gülçin Ercan, Ahmet Serdaroglu, Hatice Aygun, Oytun Erbas

**Affiliations:** 1 Department of General Surgery, Güneşli Erdem Hospital, 34212 İstanbul, Turkey; adnanhcm@hotmail.com; 2 Department of General Surgery, Faculty of Medicine, Karadeniz Technical University, 61080 Trabzon, Turkey; draakbas@hotmail.com; 3 Department of Obstetrics and Gynecology, Faculty of Medicine, Karadeniz Technical University, 61080 Trabzon, Turkey; bakiyeakbas@ktu.edu.tr; 4 Department of General Surgery, Sultan 2. Abdulhamid Han Educational and Research Hospital, University of Health Sciences, 34668 Istanbul, Turkey; ghepgul@hotmail.com; 5 Department of Internal Medicine, Faculty of Medicine, Karadeniz Technical University, 61080 Trabzon, Turkey; serdaroglu@ktu.edu.tr; 6 Neuroscience Laboratory, BAMER, Biruni University, 34010 Istanbul, Turkey; 7 Faculty of Medicine, BAMER, Biruni University, 34010 Istanbul, Turkey; oytunerbas2012@gmail.com

**Keywords:** sepsis, acute kidney injury, pioglitazone, oxidative stress, inflammation, TLR-4, NF-κB

## Abstract

**Aim**: Sepsis-associated acute kidney injury (SA-AKI) remains a major cause of mortality, driven by inflammation and oxidative stress. Pioglitazone, a PPAR-γ agonist, has demonstrated anti-inflammatory and antioxidant effects beyond glycemic control. This study evaluated its renoprotective efficacy in a rat model of sepsis induced by cecal ligation and puncture (CLP). **Methods**: Thirty-six female Wistar rats were divided into Control, CLP + Saline, and CLP + Pioglitazone (10 mg/kg/day) groups. Survival was analyzed for 5 days. Renal function (BUN, creatinine, NGAL), oxidative stress (MDA), antioxidant signaling (NRF2), and inflammatory mediators (TNF-α, IL-6, HMGB1, TLR-4, NF-κB) were quantified by ELISA. Tubular epithelial necrosis, luminal debris, dilatation, hemorrhage, and inflammation were semi-quantitatively scored. **Results**: CLP caused marked renal dysfunction with elevated BUN, creatinine, and NGAL (*p* all <0.001 vs. Control). Pioglitazone significantly reduced these markers (*p* < 0.001 vs. CLP + Saline) and improved survival. Plasma MDA levels increased and renal Nrf2 levels decreased following CLP induction (both *p* < 0.001 vs. Control), whereas pioglitazone treatment significantly reduced MDA levels and increased NRF2 expression (*p* = 0.002 and *p* < 0.001 vs. CLP + Saline, respectively). Inflammatory mediators were markedly increased in sepsis (TNF-α, IL-6, HMGB1, TLR-4, and NF-κB; all *p* < 0.001 vs. Control) and significantly downregulated by pioglitazone (*p* < 0.01, *p* < 0.001, *p* < 0.001, *p* < 0.01, *p* < 0.01 vs. CLP + Saline, respectively). Histopathological injury was pronounced in septic rats (all *p* < 0.01 vs. Control) but was markedly ameliorated by pioglitazone *p* < 0.05, indicating substantial structural recovery. **Conclusions**: Pioglitazone markedly ameliorates CLP-induced SA-AKI by suppressing TLR-4/NF-κB/TNF-α signaling and oxidative stress, improving renal structure, function, and survival. These findings support its potential repurposing as a therapeutic adjunct in sepsis management.

## 1. Introduction

Sepsis represents a critical clinical condition characterized by a maladaptive immune response to infection that precipitates systemic inflammation, circulatory collapse, and multiorgan failure. Despite advances in intensive care, it remains a major global cause of mortality, contributing to approximately one-fifth of all deaths worldwide [1,2]. Global burden analyses estimated approximately 48.9 million sepsis cases and nearly 11 million sepsis-related deaths worldwide in 2017, underscoring the substantial global impact of the disease [2]. Among affected organs, the kidney is particularly susceptible to septic injury, and the development of sepsis-associated acute kidney injury (SA-AKI) markedly worsens patient outcomes and increases morbidity and mortality [3,4,5]. Current international guidelines also emphasize that early recognition and prompt management during the first hours of sepsis are critical for improving clinical outcomes [6].

The cecal ligation and puncture (CLP) procedure is widely regarded as the most reliable experimental model for reproducing polymicrobial sepsis in animals, as it closely mimics the hemodynamic, metabolic, and immune disturbances observed in human sepsis [7,8,9,10]. In this model, fecal leakage into the peritoneal cavity triggers generalized inflammation and leads to systemic organ dysfunction similar to clinical sepsis. Recent studies have further demonstrated that the CLP model reproduces a clinically relevant spectrum of sepsis severity, including hypotension, lactate elevation, cytokine surges, and multi-organ injury patterns [11].

SA-AKI is characterized by an abrupt decline in renal function, reflected by elevated serum urea and creatinine concentrations and decreased glomerular filtration rate. The underlying pathophysiology involves intertwined mechanisms, including cytokine-driven inflammation, oxidative stress, mitochondrial impairment, and endothelial injury [12,13]. Recent consensus reports further emphasize that microcirculatory dysfunction, dysregulated immune responses, and metabolic reprogramming play key roles in SA-AKI development, even when global renal blood flow is preserved [14]. Among these, activation of the Toll-like receptor-4 (TLR-4)/nuclear factor-κB (NF-κB) signaling pathway is central, as it stimulates the release of tumor necrosis factor-α (TNF-α) and interleukin-6, promoting tubular apoptosis and microcirculatory dysfunction [15,16]. Concomitantly, reactive oxygen species-mediated lipid peroxidation exacerbates epithelial and endothelial injury [17,18]. Recent reviews also highlight emerging serum and urinary biomarkers (e.g., NGAL and HMGB1) for SA-AKI risk stratification; however, current therapeutic strategies remain largely supportive [19]. Current therapeutic approaches remain largely supportive because no pharmacological agent has yet been proven to reverse these processes effectively [20].

Pioglitazone, a peroxisome proliferator-activated receptor-γ (PPAR-γ) agonist primarily used for type 2 diabetes, exerts pleiotropic biological effects that extend beyond glycemic regulation. Its activation of PPAR-γ modulates transcriptional control of inflammation and oxidative balance, suppressing NF-κB-dependent cytokine expression while enhancing Nrf2-mediated antioxidant responses [21,22,23]. Recent mechanistic studies further highlight the crosstalk between NF-κB-driven inflammatory signaling and Nrf2-regulated antioxidant pathways, supporting therapeutic strategies that simultaneously suppress cytokine amplification and restore redox balance in sepsis-related organ injury [24]. Evidence from preclinical studies indicates that pioglitazone can attenuate TLR-4 signaling and reduce oxidative and inflammatory injury in models of hepatic and cardiac sepsis [25]. However, its efficacy as a post-treatment strategy for renal protection in polymicrobial sepsis has not yet been comprehensively examined.

Based on these considerations, the present study aimed to determine whether pioglitazone could mitigate oxidative stress and inflammatory damage in the kidneys of rats subjected to CLP-induced sepsis. To this end, renal functional markers (BUN, creatinine, NGAL), oxidative parameters (MDA), inflammatory mediators (TNF-α, TLR-4, NF-κB), and histopathological alterations were systematically evaluated.

## 2. Materials and Methods

### 2.1. Animals and Ethical Approval

A total of 36 adult female Wistar albino rats (weighing 200–250 g) were included. All procedures were conducted in compliance with the ethical guidelines of the Guide for the Care and Use of Laboratory Animals (National Institutes of Health [NIH], USA) and approved by the Institutional Animal Research Ethics Committee (Approval No: 2125095711, 28 May 2023). The animals were housed individually in polycarbonate cages under standard conditions (temperature 22 ± 2 °C, 12 h light/dark cycle) with unrestricted access to food and water. All efforts were made to minimize animal suffering, following the principles of Replacement, Reduction, and Refinement (3Rs). Statistical analyses were performed using GraphPad Prism (version 9.0, GraphPad Software, San Diego, CA, USA) and IBM SPSS Statistics (version 25.0/IBM Corp./Armonk, NY, USA).

### 2.2. Experimental Design

Rats were randomly assigned into three experimental groups (*n* = 12 per group):

Control: sham-operated rats without cecal manipulation, maintained on standard diet and water.

CLP + Saline: sepsis induced by cecal ligation and puncture (CLP), followed by intraperitoneal injection of physiological saline (0.9% NaCl).

CLP + Pioglitazone: CLP-induced sepsis treated with pioglitazone (10 mg/kg/day, intraperitoneally).

In the CLP + Pioglitazone group, sepsis was induced by cecal ligation and puncture, and pioglitazone was administered intraperitoneally at a dose of 10 mg/kg. The first injection was performed 1 h after the CLP surgery, followed by subsequent injections once every 24 h during the 5-day experimental period. This post-treatment regimen was designed to evaluate the therapeutic efficacy of pioglitazone rather than its prophylactic potential in sepsis-associated acute kidney injury.

Fluid resuscitation was administered equally in septic groups (30 mL/kg 0.9% NaCl within the first postoperative hour, followed by 10 mL/kg/day in divided doses). The experiment continued for five days; during this period, six rats from the CLP + Saline and two from the CLP + Pioglitazone group did not survive.

**Figure 1:**
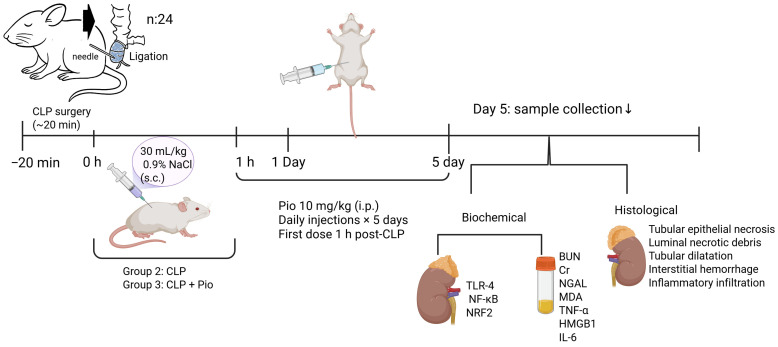
Schematic representation of the experimental design and procedures. Polymicrobial sepsis was induced by cecal ligation and puncture (CLP) under anesthesia. Rats received fluid resuscitation (30 mL/kg, 0.9% NaCl, s.c.) immediately after CLP. Pioglitazone (10 mg/kg, i.p.) was administered 1 h after surgery and repeated once daily for five days. On Day 5, blood and kidney samples were collected for biochemical (BUN, creatinine, NGAL, MDA, TNF-α, TLR-4, NF-Κb, HMGB1, IL-6, NRF2) and histopathological (tubular epithelial necrosis, luminal necrotic debris, tubular dilatation, interstitial hemorrhage, inflammatory infiltration) analyses.

At the study endpoint, surviving animals were anesthetized with ketamine/xylazine, and blood samples were obtained by cardiac puncture. Both kidneys were excised for biochemical and histopathological analyses (Figure 1).

### 2.3. Cecal Ligation and Puncture (CLP) Procedure

Under aseptic conditions and general anesthesia, a midline laparotomy (~3 cm) was performed to expose the cecum. The distal third of the cecum was ligated using 3–0 silk suture approximately 1 cm from the ileocecal junction to prevent intestinal obstruction, and the cecal wall was punctured once with a 22-gauge sterile needle. A small amount of fecal material was gently extruded to confirm perforation before the cecum was repositioned and the abdominal wall was closed in two layers with 4–0 absorbable sutures.

Sham-operated animals underwent laparotomy without ligation or puncture. In accordance with previously described CLP protocols, systemic sepsis was induced following the procedure [26].

### 2.4. Histopathological Examination of the Kidney

At the endpoint, both kidneys were carefully removed, rinsed with cold saline, and immersed in 10% neutral buffered formalin (pH 7.4) for 72 h. Following fixation, tissues were processed using standard paraffin-embedding techniques and sectioned at 5 µm thickness. The sections were stained with hematoxylin and eosin (H&E) for morphological evaluation under a light microscope (Olympus BX51, Tokyo, Japan).

Digital images were captured from ten randomly selected non-overlapping cortical fields (×20 magnification) using an integrated imaging system (Image-Pro Express, Media Cybernetics, Rockville, MD, USA). A pathologist blinded to experimental grouping performed semi-quantitative scoring of histological changes, including tubular epithelial necrosis, luminal debris accumulation, tubular dilatation, interstitial hemorrhage, and inflammatory cell infiltration.

Each parameter was graded according to the percentage of affected cortical area as follows:

0 = 0–5%, 1 = 6–20%, 2 = 21–40%, 3 = 41–60%, 4 = 61–80%, and 5 = 81–100% of tissue involvement, modified from Erbas et al. [26].

### 2.5. Biochemical Measurements

#### 2.5.1. Plasma Biomarkers

After euthanasia, blood was collected into heparinized tubes via cardiac puncture. Samples were centrifuged at 3000 rpm for 10 min, and plasma was separated and stored at −20 °C until analysis. Blood urea nitrogen (BUN) and creatinine concentrations were determined using an automated chemistry analyzer (Beckman Coulter AU640, Brea, CA, USA) and standard commercial assay kits.

Plasma levels of tumor necrosis factor-α (TNF-α), neutrophil gelatinase-associated lipocalin (NGAL) and IL-6 were quantified using rat-specific enzyme-linked immunosorbent assay (ELISA) kits (Abcam, UK), performed in duplicate according to the manufacturers’ protocols. HMGB1 levels were measured using a commercially available ELISA kit (IBL International, Hamburg, Germany, Cat. No. ST51011). Optical densities were read at 450 nm using a microplate spectrophotometer.

#### 2.5.2. Renal Tissue Assays

Kidney samples were homogenized in ice-cold phosphate-buffered saline (pH 7.4) at a ratio of 1:5 (*w*/*v*). Homogenates were centrifuged at 5000× *g* for 15 min at 4 °C, and supernatants were collected for biochemical analyses. Total protein content was determined by the Bradford method using bovine serum albumin as the calibration standard.

Levels of toll-like receptor 4 (TLR-4) and nuclear factor-κB (NF-κB) in renal tissue were quantified using commercial rat-specific ELISA kits (Abcam, Waltham, MA, USA). Nuclear factor erythroid 2–related factor 2 (Nrf2) levels in tissue homogenates were measured using a rat-specific quantitative sandwich ELISA kit (MyBioSource, MBS1602926, San Diego, CA, USA) according to the manufacturer’s instructions. Lipid peroxidation was evaluated by measuring malondialdehyde (MDA) using the thiobarbituric acid reactive substances (TBARS) method. Briefly, samples were incubated with trichloroacetic acid and thiobarbituric acid at 100 °C for 1 h, cooled on ice, centrifuged, and the absorbance of the supernatant was measured at 535 nm. MDA concentrations were expressed as nmol/mL using 1,1,3,3-tetraethoxypropane as the calibration standard.

### 2.6. Statistical Analysis

Data distribution was assessed using the Shapiro–Wilk test for normality and Levene’s test for homogeneity of variances. Normally distributed data were analyzed using one-way ANOVA, followed by Tukey’s or Tamhane’s T2 post hoc tests where appropriate. Non-parametric data were expressed as median [interquartile range, IQR] and compared using the Kruskal–Wallis test, followed by Mann–Whitney U for pairwise analyses. Kaplan–Meier survival analysis was conducted with the log-rank (Mantel–Cox) test. All statistical analyses were performed using SPSS version 25.0 (IBM Corp., Armonk, NY, USA), and a *p* value < 0.05 was considered statistically significant.

## 3. Results

### 3.1. Survival Analysis

**Figure 2:**
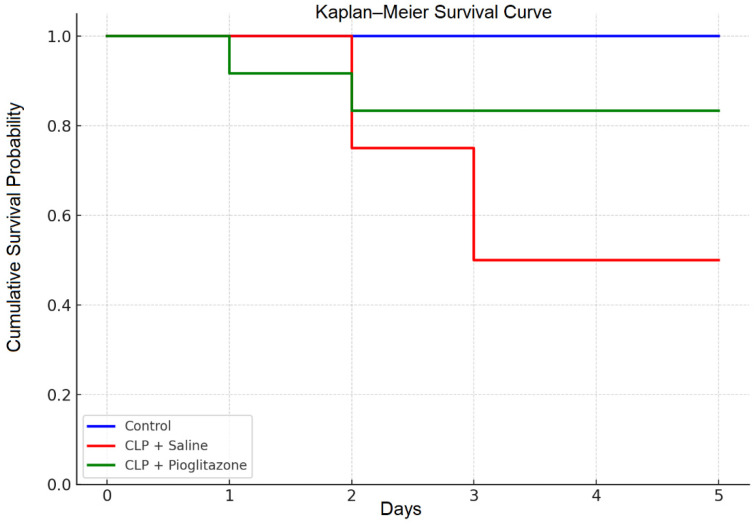
Kaplan–Meier survival curves of experimental groups. Pioglitazone (10 mg/kg, i.p.) markedly improved 5-day survival (83.3%) compared with the CLP + saline group (50%), approaching control levels (100%).

Kaplan–Meier survival analysis demonstrated statistically significant differences among the experimental groups (Log-rank test: χ^2^ = 8.97, df = 2, *p* = 0.011). The CLP + saline group exhibited a substantial reduction in 5-day survival (50%), while pioglitazone treatment markedly improved survival to 83.3%, closely approaching the 100% survival observed in the non-septic control group. These results suggest that pioglitazone provides a significant protective effect in this rat sepsis model (Figure 2, Table 1).

The table summarizes the number of surviving rats on each experimental day. Pioglitazone treatment improved overall survival compared with the CLP + saline group.

### 3.2. Renal Function and Histopathology

#### 3.2.1. Quantitative Histopathological Scores

**Figure 3:**
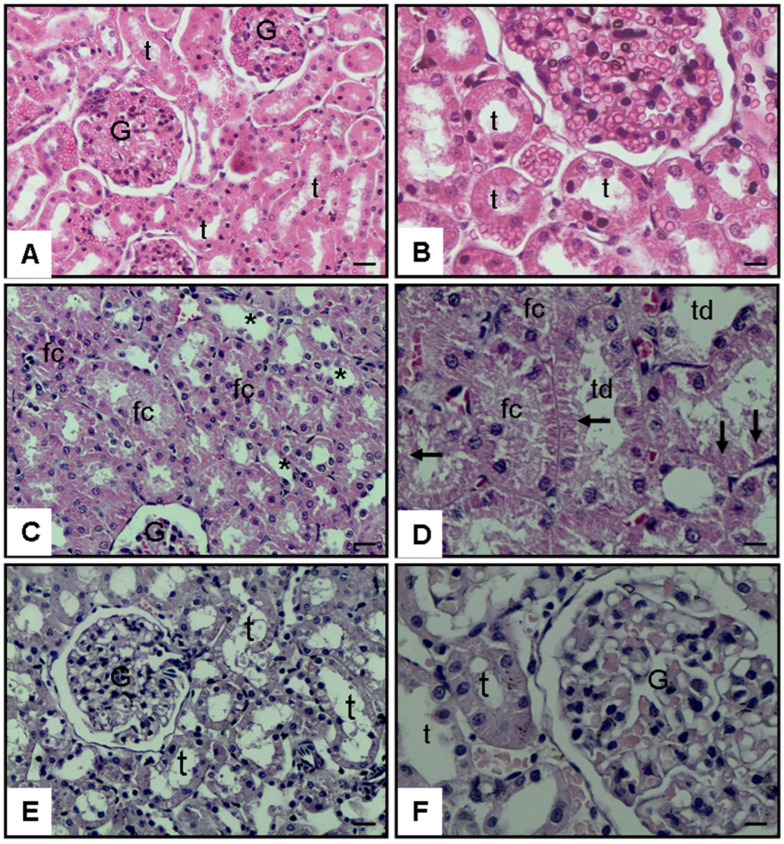
Kidney histopathology ×20 and ×40 magnification, hematoxylin and eosin (H&E) stain. (**A**,**B**): Normal group kidney, renal tubules (t); Glomerulus (G). (**C**,**D**): CLP and saline groups showed severe histopathologic alterations with tubular dilatation (td), tubular epithelial necrosis (arrow), and luminal fibrin cast (fc). (**E**,**F**): CLP and 10 mg/kg pioglitazone groups showed decreased injury with nearly normal tubules and tubular epithelial cells (t).

**Figure 4:**
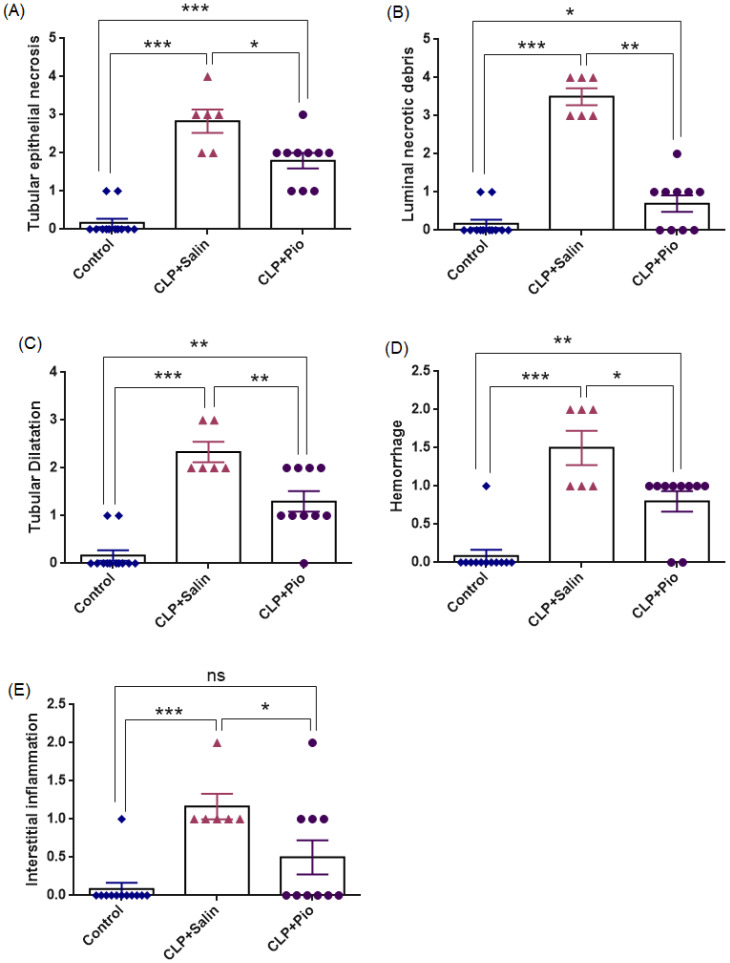
Histopathological scoring of renal injury parameters in CLP-induced sepsis and the protective effect of pioglitazone. Data show (**A**) tubular epithelial necrosis, (**B**) luminal necrotic debris, (**C**) tubular dilatation, (**D**) hemorrhage, and (**E**) interstitial inflammation across groups (Control, CLP + Saline, CLP + Pioglitazone). (*n* = 6–10). * *p* < 0.05, ** *p* < 0.01, *** *p* < 0.001; ns = not significant.

Representative histopathological findings are presented in Figure 3 and Figure 4 and Table 2, demonstrating that CLP-induced sepsis caused extensive renal structural injury, whereas pioglitazone treatment markedly alleviated these alterations.

Histological examination revealed intact corticomedullary architecture in the control group. In contrast, the CLP + Saline group exhibited prominent tubular epithelial necrosis, luminal necrotic debris accumulation, tubular dilatation, hemorrhage, and interstitial inflammation (overall Kruskal–Wallis *p* < 0.001; pairwise comparisons *p* < 0.001), indicating marked tubular injury accompanied by microvascular bleeding, leukocyte infiltration, and tissue inflammation after sepsis induction. Pioglitazone treatment significantly ameliorated these lesions (*p* = 0.016; 0.001; 0.009; 0.017; and 0.042 vs. CLP + Saline, respectively), although complete normalization was not achieved. Tubular epithelial necrosis (*p* < 0.001), luminal necrotic debris (*p* = 0.035), tubular dilatation (*p* = 0.001), and hemorrhage (*p* = 0.017) scores remained higher than in controls, suggesting partial yet meaningful structural recovery rather than full restoration. Pioglitazone group did not differ significantly from Control (*p* = 0.078), suggesting that inflammation levels were nearly normalized after treatment, indicating a strong trend toward complete restoration of tissue inflammatory status to baseline levels.

#### 3.2.2. Representative Micrographs of Renal Injury 

Histopathological evaluation of kidney sections revealed marked differences among the experimental groups. The control group exhibited normal renal morphology with intact glomeruli and tubular structures (Figure 3A,B). In contrast, the CLP + Saline group demonstrated extensive tubular epithelial necrosis, luminal necrotic debris, tubular dilatation, and interstitial inflammatory cell infiltration (Figure 3C,D). Tubular epithelial flattening and focal vascular congestion were also evident, indicating severe sepsis-induced renal injury. Treatment with pioglitazone notably alleviated these pathological alterations, as reflected by preserved tubular integrity, reduced inflammatory infiltration, and nearly normal glomerular appearance (Figure 3E,F). These findings corroborate the quantitative histopathological scores, confirming the renoprotective effect of pioglitazone against CLP-induced septic damage.

#### 3.2.3. Renal Function Biomarkers

**Figure 5:**
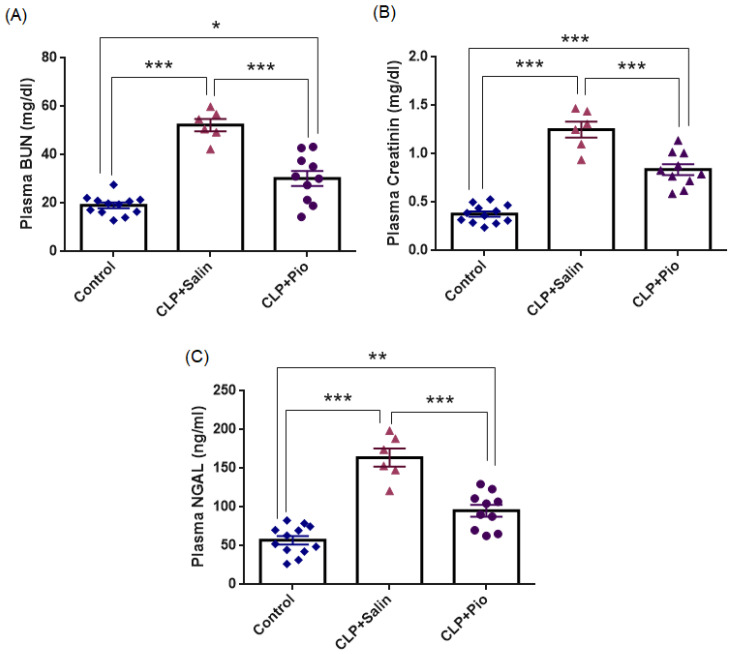
Plasma renal function markers in experimental groups. (**A**) Plasma blood urea nitrogen (BUN), (**B**) plasma creatinine, and (**C**) plasma neutrophil gelatinase-associated lipocalin (NGAL) level across groups. CLP + Saline induced marked elevations, while pioglitazone significantly reduced these increases. * *p* < 0.05, ** *p* < 0.01, *** *p* < 0.001.

To evaluate the effect of CLP-induced sepsis on renal injury across groups, plasma BUN, creatinine, and NGAL levels were quantified. As shown in Figure 5 and Table 3, the CLP + Saline group exhibited significantly higher BUN, creatinine, and NGAL values than the control group (all *p* < 0.001). When the mean control value was taken as the baseline, the CLP + Saline group showed 2.73-fold, 3.38-fold, and 2.87-fold increases in BUN, creatinine, and NGAL, respectively. Pioglitazone treatment markedly attenuated these elevations (all *p* < 0.001 vs. CLP + Saline), corresponding to reductions of 42.1%, 33.6%, and 41.9%, respectively. Nevertheless, values remained modestly above baseline (BUN *p* = 0.018; creatinine *p* < 0.001; NGAL *p* = 0.002), indicating partial but meaningful recovery of renal function. Collectively, the biochemical improvements in renal function biomarkers (BUN, creatinine, and NGAL) were consistent with the histopathological recovery, indicating that pioglitazone substantially mitigated sepsis-induced acute tubular injury.

### 3.3. Oxidative Stress and Inflammatory Biomarkers

#### 3.3.1. Oxidative Stress Biomarker

**Figure 6:**
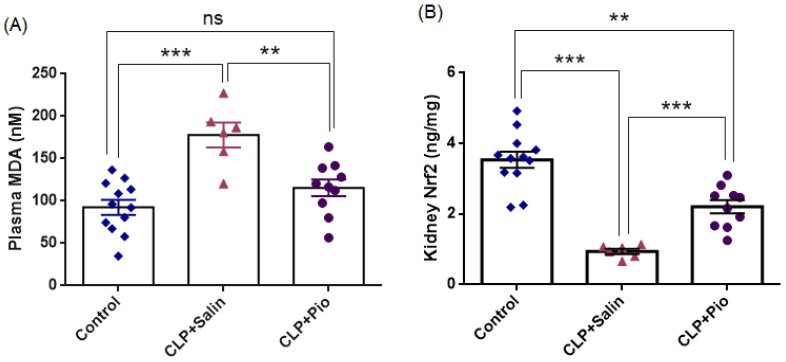
Pioglitazone attenuates sepsis-induced oxidative stress and restores antioxidant signaling. (**A**) Plasma MDA and (**B**) kidney Nrf2 levels across experimental groups (Control, CLP + Saline, CLP + Pio). Bars represent mean ± SEM with individual animal values (Control *n* = 12; CLP + Saline *n* = 6; CLP + Pio *n* = 10). Statistical significance was determined by one-way ANOVA followed by Tukey’s multiple comparisons test. ** *p* < 0.01, *** *p* < 0.001; ns, not significant.

An overview of the oxidative stress findings is presented in Figure 6 and Table 4, showing that CLP-induced sepsis markedly increased systemic lipid peroxidation and suppressed renal antioxidant signaling, whereas pioglitazone treatment substantially attenuated lipid peroxidation and partially improved antioxidant responses.

Plasma MDA levels were significantly higher in the CLP + Saline group (177.9 ± 14.6 nmol/mL) than in the Control group (92.5 ± 8.8 nmol/mL; *p* < 0.001), representing an approximately 1.9-fold increase in lipid peroxidation following sepsis induction. Pioglitazone treatment markedly reduced MDA concentrations (*p* < 0.01 vs. CLP + Saline), corresponding to an estimated 35% decrease relative to untreated sepsis. Importantly, MDA values in the pioglitazone group were statistically comparable to controls (*p* = 0.231), indicating near-complete restoration of oxidative balance.

Collectively, these findings suggest that pioglitazone mitigated sepsis-induced oxidative damage, likely by limiting lipid peroxidation and enhancing redox balance.

Renal Nrf2 levels were significantly lower in the CLP + Saline group (0.942 ± 0.074 ng/mL) than in the Control group (3.537 ± 0.229 ng/mL; *p* < 0.001), representing an approximately 3.8-fold decrease in antioxidant signaling following sepsis induction. Pioglitazone treatment markedly increased NRF2 concentrations (*p* < 0.01 vs. CLP + Saline), corresponding to an approximately 2.3-fold increase relative to untreated sepsis. Importantly, NRF2 values in the pioglitazone group remained significantly lower than those observed in controls (*p* < 0.01), indicating partial improvement of antioxidant defense.

Collectively, these findings suggest that pioglitazone mitigated sepsis-induced renal oxidative damage, likely by modulating NRF2-mediated antioxidant signaling and improving renal redox balance.

#### 3.3.2. Inflammatory Biomarkers

**Figure 7:**
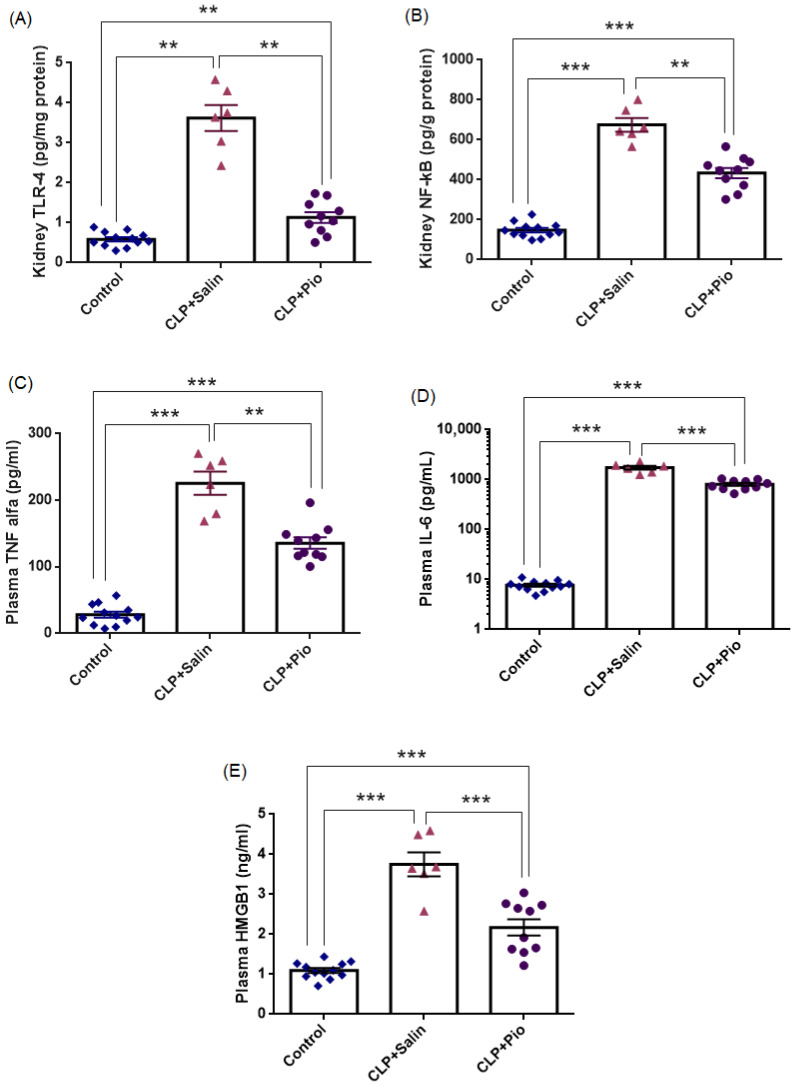
Pioglitazone attenuates sepsis-induced inflammatory responses. (**A**) Kidney TLR-4, (**B**) kidney NF-κB, (**C**) plasma TNF-α, (**D**) plasma IL-6, and (**E**) plasma HMGB1 levels across experimental groups (Control, CLP + Saline, CLP + Pio). Bars represent mean ± SEM with individual animal values (Control *n* = 12; CLP + Saline *n* = 6; CLP + Pio *n* = 10). Statistical significance was determined using one-way ANOVA followed by Tukey’s multiple comparisons test. For IL-6 (panel D), the *y*-axis is presented on a logarithmic scale to facilitate visualization of the wide range of cytokine concentrations. ** *p* < 0.01, *** *p* < 0.001.

Figure 7 and Table 4 summarize the principal inflammatory findings, demonstrating marked activation of innate inflammatory responses following CLP-induced sepsis, which were substantially attenuated by pioglitazone treatment. As shown in Figure 7, the CLP + Saline group exhibited significant up-regulation of TLR-4, NF-κB, and TNF-α and a marked elevation of plasma IL-6 compared with the Control group (*p* < 0.01; *p* < 0.001; *p* < 0.001; and *p* < 0.001, respectively). Relative to baseline, TLR-4, NF-κB, and TNF-α levels increased approximately 6.2-fold, 4.6-fold, and 8.0-fold, respectively, while plasma IL-6 increased approximately 226-fold, indicating a robust activation of the TLR-4/NF-κB signaling axis and a pronounced systemic inflammatory response characteristic of sepsis.

Pioglitazone treatment markedly attenuated these elevations (all *p* < 0.001 vs. CLP + Saline), corresponding to reductions of 69%, 36%, and 40%, respectively. Similarly, plasma IL-6 levels were substantially reduced in the CLP + Pio group compared with the CLP + Saline group, representing an approximate 54% decrease (*p* < 0.001).

Despite this marked attenuation, values in the pioglitazone group remained significantly higher than those observed in the Control group (*p* < 0.01; *p* < 0.001; *p* < 0.001; and *p* < 0.001, respectively), indicating partial yet biologically meaningful suppression of inflammation. Collectively, these findings demonstrate that pioglitazone downregulated renal TLR-4 and NF-κB expression and reduced circulating TNF-α and IL-6 levels, thereby mitigating the excessive inflammatory and cytokine responses characteristic of sepsis.

As shown in Figure 7, the CLP + Saline group exhibited significantly higher HMGB1 levels than the control group (*p* < 0.001). When the mean control value was taken as the baseline, the CLP + Saline group showed an approximately 3.43-fold increase in plasma HMGB1 concentrations. Pioglitazone treatment markedly attenuated this elevation (*p* < 0.001 vs. CLP + Saline), corresponding to a 42.0% reduction relative to untreated sepsis. Nevertheless, HMGB1 levels remained significantly higher than the control values (*p* = 0.002), indicating partial but meaningful suppression of systemic inflammatory activation. Collectively, these findings suggest that pioglitazone substantially mitigated sepsis-associated inflammatory signaling reflected by circulating HMGB1 levels.

## 4. Discussion

In the present study, we demonstrated that pioglitazone, a PPAR-γ agonist, significantly alleviates sepsis-induced acute kidney injury (SA-AKI) by suppressing TLR-4/NF-κB–mediated inflammation and oxidative stress, thereby improving renal structure, function, and survival. Within this framework, activation of PPARγ may represent a promising therapeutic strategy for mitigating sepsis-associated renal injury.

Sepsis was induced via cecal ligation and puncture (CLP), a well-established model that replicates the hemodynamic, immunological, and microbial complexity of human polymicrobial sepsis [7]. Recent methodological analyses note that CLP severity and organ injury are highly sensitive to surgical parameters and supportive care conditions, highlighting the importance of standardized protocols for reproducibility [27]. Moreover, graded CLP paradigms with longitudinal phenotyping have been proposed to better capture clinically relevant heterogeneity ranging from moderate illness to fulminant septic shock [11]. In this model, the leakage of fecal material into the peritoneal cavity produces polymicrobial peritonitis, which precipitates a systemic inflammatory response and subsequent multiorgan dysfunction. Model validity was confirmed by marked increases in plasma BUN, creatinine, and NGAL levels, accompanied by the classic histopathological features of acute tubular necrosis—tubular epithelial necrosis, luminal debris, tubular dilatation, interstitial hemorrhage, and inflammatory infiltration—consistent with prior CLP-based sepsis studies [28,29,30,31].

In the present study, pioglitazone treatment markedly attenuated renal dysfunction, as evidenced by normalization of BUN and creatinine levels and a significant reduction in NGAL, a sensitive biomarker that increases earlier than creatinine during early tubular injury [32,33,34]. Contemporary SA-AKI frameworks emphasize that creatinine-based functional criteria may lag behind early tubular stress, and therefore recommend the combined interpretation of functional parameters with damage biomarkers such as NGAL [14]. However, NGAL levels may also rise in sepsis in the absence of overt AKI due to systemic inflammatory activation, highlighting the importance of interpreting NGAL in conjunction with creatinine dynamics and the broader inflammatory context [32,35]. Accordingly, the recent literature increasingly supports multi-biomarker approaches rather than reliance on a single marker for a more accurate characterization of kidney injury [36]. In this context, the reduction in NGAL observed in our study, together with the improvement in BUN and creatinine levels and the marked attenuation of histopathological features such as tubular injury and inflammatory infiltration, suggests that pioglitazone exerts both functional and structural protective effects against sepsis-associated renal injury.

This biochemical recovery was paralleled by a survival benefit: mortality decreased from 50% in untreated septic rats to 16.7% with pioglitazone administration. These findings align with previous reports showing that PPAR-γ activation improves survival and reduces systemic inflammation during sepsis. For instance, pioglitazone pretreatment was shown to lower IL-6 and MCP-1 expression while increasing 7-day survival [37], whereas prolonged administration decreased TNF-α and IL-1β levels and improved outcomes in severe sepsis [38]. Together with our results, these studies reinforce the notion that pioglitazone mitigates the systemic inflammatory injury that drives SA-AKI-related mortality.

CLP-induced sepsis markedly increased renal TLR-4 and NF-κB expression together with elevated TNF-α and IL-6, indicating activation of innate immune signaling in the kidney. Consistent with CLP-based sepsis models, robust activation of the TLR-4/NF-κB inflammatory axis during sepsis-associated AKI has been widely reported [39,40,41,42]. In addition to classical cytokines, the damage-associated molecular pattern HMGB1 has been implicated in the regulation of inflammatory signaling during sepsis. HMGB1 released during tissue injury activates TLRs and RAGE receptors, amplifying NF-κB–driven inflammatory signaling and contributing to renal injury in SA-AKI [43,44]. Consistent with this mechanism, circulating IL-6, TNF-α and HMGB1 levels were elevated in the CLP group, reflecting systemic inflammatory activation.

Pioglitazone significantly reduced renal TLR-4 and NF-κB expression together with decreased TNF-α and IL-6 levels, indicating suppression of this pro-inflammatory cascade. These findings agree with previous studies showing that pioglitazone downregulates TLR-4/MyD88 signaling, inhibits NF-κB nuclear translocation, and stabilizes IκB-α, ultimately reducing systemic TNF-α and IL-6 release [21,37]. In parallel, pioglitazone also reduced circulating HMGB1 levels, suggesting attenuation of DAMP-mediated inflammatory amplification. Upregulation of adiponectin, a downstream effector of PPAR-γ, may further suppress NF-κB activity and reinforce this anti-inflammatory effect [21]. Although our measurements reflect protein levels rather than direct pathway activation, the findings are consistent with attenuation of the HMGB1/TLR4/NF-κB inflammatory axis [45].

IL-6 is an early biomarker of sepsis and correlates with disease severity in ICU cohorts [46]. In the present study, the concurrent reduction in IL-6 and HMGB1 following pioglitazone treatment further supports its systemic immunomodulatory effect. Future studies should evaluate anti-inflammatory cytokines such as IL-10 to better characterize sepsis-associated immune dysregulation and its potential links to tumor immune escape [47].

In addition to inflammation, oxidative stress plays a key role in the pathogenesis of sepsis. Excessive production of reactive oxygen species (ROS) during systemic inflammation promotes lipid peroxidation, resulting in malondialdehyde (MDA) accumulation and cellular injury [48]. Clinical observations also suggest that oxidative stress biomarkers such as MDA dynamically change during early sepsis and may reflect developing organ dysfunction [49]. Consistent with these reports, MDA levels were markedly elevated in the CLP + Saline group in the present study, indicating pronounced oxidative stress in sepsis-associated AKI. Similar increases in plasma and renal MDA have been reported in experimental CLP models [50,51,52,53].

Parallel to its anti-inflammatory effects, pioglitazone exerted a significant antioxidant action, as evidenced by reduced plasma MDA levels and partial restoration of renal Nrf2 protein suppressed by CLP. Nrf2 is a central regulator of antioxidant defense and plays a protective role in sepsis-related organ injury, including septic AKI [54]. Consistent with previous studies showing that pioglitazone reduces oxidative stress markers such as MDA and myeloperoxidase (MPO), these findings suggest attenuation of neutrophil-mediated oxidative injury [25,55].

Mechanistically, our results support a PPARγ-linked redox regulatory pathway. CLP-induced sepsis decreased renal Nrf2 protein, whereas pioglitazone partially restored Nrf2 toward control values. Nrf2 signaling interacts with inflammatory pathways through reciprocal antagonism with NF-κB, potentially explaining the concurrent reduction in inflammatory cytokines and oxidative damage observed in the present study [24]. In addition, experimental evidence indicates that PPARγ activation can enhance Nrf2 signaling in kidney injury models, supporting a mechanistic link between pioglitazone treatment and restoration of antioxidant defense [56].

Histopathological examination confirmed the biochemical findings, showing that pioglitazone markedly reduced tubular necrosis, debris accumulation, interstitial inflammation, and hemorrhage, restoring near-normal renal architecture. The parallel structural and functional recovery underscores its renoprotective efficacy. Comparable effects observed with other TLR-4/NF-κB inhibitors, such as paeonol in endotoxin-induced AKI [57], validate this pathway as a common therapeutic target in septic renal injury. By simultaneously suppressing TLR-4/NF-κB-driven inflammation and oxidative stress, pioglitazone disrupts the cytokine–ROS cycle underlying multiorgan dysfunction, preserving renal function and improving survival.

The translational relevance lies in the potential repurposing of pioglitazone for sepsis—an area that still urgently requires effective adjunctive therapies. As an U.S. Food and Drug Administration approved antidiabetic drug with a well-characterized clinical safety profile, pioglitazone represents a practical repurposing candidate. Epidemiological data indicate lower sepsis-related mortality among chronic users (OR = 0.80; ref. [58]), and phase I studies have confirmed feasible administration in critically ill patients [59]. Recent immunotherapy frameworks further emphasize that successful sepsis interventions will likely require phenotype-guided and carefully timed immunomodulation rather than uniform anti-inflammatory strategies [60]. Importantly, sepsis is a major complication in immunocompromised oncology populations—particularly after cytotoxic chemotherapy where febrile neutropenia/neutropenic sepsis remains associated with substantial ICU mortality and distinct risk profiles across cancer subtypes [61]. In addition, septic AKI is clinically relevant in cancer patients with sepsis and is linked to shock severity and organ-failure burden, strengthening the need for renoprotective adjuncts that can be deployed without compromising antimicrobial priorities [62]. Beyond infection-driven organ failure, chemotherapy-induced AKI remains a key limitation to treatment intensity in cancer care [63], and pioglitazone’s anti-inflammatory/antioxidant mechanisms (TLR4/NF κB suppression and restoration of cytoprotective redox programs) may therefore be mechanistically relevant to kidney vulnerability in oncology. At the same time, immunometabolic targeting of PPARγ is actively explored in cancer biology and therapy [64]; notably, pioglitazone has been reported to reduce tumor PD L1 protein and to enhance anti PD 1 efficacy in preclinical colorectal cancer models [65], whereas tumor-cell PPARγ signaling has also been implicated in immune evasion and resistance to immune-checkpoint blockade in other contexts, highlighting that the direction and timing of PPARγ modulation may be tumor- and setting-dependent [66]. Finally, potential oncologic safety signals should be acknowledged: evidence regarding pioglitazone and bladder cancer remains mixed and appears most relevant to long-term exposure, so short-course use as an acute sepsis adjunct would be expected to entail a different risk profile but warrants caution in patients with prior urothelial malignancy or unexplained hematuria [67,68].

Long-standing male bias in preclinical research has resulted in substantial sex underrepresentation, with male-only designs remaining common across biomedical disciplines [69,70]. Quantitative analyses further show that inclusion of females does not inherently increase experimental variability, even without strict estrous-cycle control [71,72]. Sex differences in sepsis and acute kidney injury (AKI) appear model-dependent. While sex effects are evident in some inflammatory injury settings, CLP-based polymicrobial sepsis models often show limited differences in early survival or organ injury [73], and clinical studies similarly report comparable SA-AKI incidence between men and women after adjustment [74,75]. Accordingly, the use of female rats in this study served as an initial proof-of-concept evaluation in an underrepresented sex, although confirmation in both sexes is warranted. Estrous-cycle stage was not monitored. Because estrogen can influence inflammatory signaling, including NF-κB-related pathways, hormonal variation may affect responses and should be addressed in future studies [76]. In addition, sex-related differences in immune responses and drug metabolism—including variation in CYP450 activity relevant to anticancer therapies—may influence translational outcomes in oncology populations [77,78,79].

To ensure adequate PPARγ target engagement in polymicrobial sepsis, we selected pioglitazone at 10 mg/kg/day based on prior CLP studies showing that post-treatment PPARγ activation attenuates NF-κB signaling and systemic inflammation [21], as well as oncology-relevant models demonstrating reduced NF-κB activation and oxidative stress at the same dose [80]. The first dose was administered 1 h after CLP induction to model a clinically relevant post-treatment scenario reflecting early therapeutic intervention after the onset of sepsis. Early bacteremia and endotoxemia have been reported within the first hours following CLP, supporting early post-treatment designs in experimental sepsis studies [81,82].

We administered pioglitazone for a short course (5 days), consistent with a phase-1 sepsis pharmacokinetic and safety study [59], which may limit duration-dependent adverse effects such as fluid retention. Because PPARγ agonists can promote fluid retention and hemodynamic stress at higher exposures, careful dose optimization will be important for future translational applications, particularly in oncology populations. Importantly, epidemiological concerns regarding pioglitazone and bladder cancer have primarily been reported in the context of long-term cumulative exposure [83,84]. In contrast, short-course administration in acute inflammatory conditions such as sepsis is unlikely to involve comparable exposure profiles.

### Limitation

In this study, a single dose derived from prior efficacy studies was employed rather than a full dose–response design. Although this approach demonstrated consistent effects on survival, renal biomarkers, and inflammatory and oxidative endpoints in our CLP-SA-AKI post-treatment model, it does not allow identification of the minimal effective dose. Therefore, future studies should evaluate lower doses, characterize pharmacokinetic profiles in septic versus non-septic hosts, and monitor potential adverse effects such as fluid retention, which may be clinically relevant for oncology populations [85,86,87].

We did not assess emerging renal stress biomarkers such as urinary [TIMP-2]·[IGFBP7] or circulating cell-free DNA, which have been highlighted in recent AKI and sepsis-AKI biomarker literature; incorporating these panels may strengthen oncology-relevant phenotyping in future studies [88,89,90]. In addition, downstream metabolic targets of PPAR-γ activation, such as CD36 and GLUT4, were not evaluated in the present study, and their assessment may further clarify on-target pharmacological effects of pioglitazone in septic AKI.

## 5. Conclusions

In this study, findings establish that pioglitazone confers potent renoprotective, anti-inflammatory, and antioxidant effects in sepsis-associated AKI. By downregulating TLR-4/NF-κB signaling and mitigating oxidative stress, the compound interrupts the cytokine–redox cycle that drives multiorgan failure, leading to improved renal structure, function, and survival.

## Figures and Tables

**Table 1:** Daily survival counts of experimental groups after CLP.

	Baseline (*n*)	Day 1	Day 2	Day 3	Day 4	Day 5
Control	12	12	12	12	12	12
CLP + Salin	12	12	9	6	6	6
CLP + Pio	12	11	10	10	10	10

**Table 2:** Histopathological scoring parameters in CLP-induced sepsis and the effect of pioglitazone treatment.

	Control	CLP + Saline	CLP + Pio
Tubular epithelial necrosis	0.0 [0.0–0.0]	3.0 [2.0–3.25] ^a^	2.0 [1.0–2] ^b^
Luminal necrotic debris	0.0 [0.0–0.0]	3.5 [3.0–4.0] ^a^	1.0 [0.0–1.0] ^c^
Tubular Dilatation	0.0 [0.0–0.0]	2.0 [2.0–3.0] ^a^	1.0 [1.0–2.0] ^c^
Hemorrhage	0.0 [0.0–0.0]	1.5 [1.0–2.0] ^a^	1.0 [0.75–1.0] ^b^
Interstitial inflammation	0.0 [0.0–0.0]	1.0 [1.0–1.25] ^a^	0.5 [0.0–1.0] ^b^

Superscript letters indicate statistical significance: a *p* < 0.001 vs. Control; b *p* < 0.05, c *p* < 0.01 vs. CLP + Saline.

**Table 3:** Renal function biomarkers across experimental groups.

	Control (*n* = 12)	CLP + Saline (*n* = 6)	CLP + Pio (*n* = 10)
Plasma BUN (mg/dL)	19.1 ± 1.1	52.2 ± 2.5 ^a^	30.2 ± 3.1 ^b^
Plasma Creatinine (mg/dL)	0.37 ± 0.02	1.25 ± 0.08 ^a^	0.83 ± 0.05 ^b^
Plasma NGAL (ng/mL)	57.2 ± 5.4	163.9 ± 11.7 ^a^	95.2 ± 7.5 ^b^

Superscript letters indicate significance levels: a *p* < 0.001 vs. Control group; b *p* < 0.001 vs. CLP + Saline group.

**Table 4:** Oxidative stress and inflammatory markers across experimental groups.

	Control (*n* = 12)	CLP + Saline (*n* = 6)	CLP + Pio (*n* = 10)
Plasma MDA (nM)	92.5 ± 8.8	177.9 ± 14.6 ^b^	115.6 ± 9.8 ^c^
NRF2 (ng/mg)	3.537 ± 0.229	0.942 ± 0.074 ^b^	2.206 ± 0.185 ^a,d^
Kidney TLR-4 (pg/mg protein)	0.58 ± 0.05	3.62 ± 0.32 ^a^	1.13 ± 0.14 ^a,c^
Kidney NF-κB (pg/g protein)	148.2 ± 10.8	675.2 ± 34.79 ^b^	433.8 ± 26.16 ^b,c^
Plasma TNF-α (pg/mL)	28.17 ± 4.4	225.7 ± 17.4 ^b^	135.7 ± 8.6 ^b,c^
Plasma IL-6 (pg/mL)	7.72 ± 0.50	1744 ± 152.9 ^b^	804.6 ± 55.25 ^b,d^
HMGB1 (ng/mL)	1.09 ± 0.06	3.75 ± 0.29 ^b^	2.17 ± 0.20 ^b,d^

Superscript letters indicate statistical significance: a *p* < 0.01, b *p* < 0.001 vs. Control; c *p* < 0.01, d *p* < 0.001 vs. CLP + Saline.

## Data Availability

The data that support the findings of this study are not publicly available due to ethical reasons but are available from the corresponding author upon request.
